# Advanced Technologies for Local Neural Circuits in the Cerebral Cortex

**DOI:** 10.3389/fnana.2021.757499

**Published:** 2021-11-03

**Authors:** Masaaki Endo, Hisato Maruoka, Shigeo Okabe

**Affiliations:** Department of Cellular Neurobiology, Graduate School of Medicine and Faculty of Medicine, The University of Tokyo, Tokyo, Japan

**Keywords:** local neural circuit, optical clearing, expansion microscopy, volumetric electron microscopy, spatial transcriptomics, lineage tracing

## Abstract

The neural network in the brain can be viewed as an integrated system assembled from a large number of local neural circuits specialized for particular brain functions. Activities of neurons in local neural circuits are thought to be organized both spatially and temporally under the rules optimized for their roles in information processing. It is well perceived that different areas of the mammalian neocortex have specific cognitive functions and distinct computational properties. However, the organizational principles of the local neural circuits in different cortical regions have not yet been clarified. Therefore, new research principles and related neuro-technologies that enable efficient and precise recording of large-scale neuronal activities and synaptic connections are necessary. Innovative technologies for structural analysis, including tissue clearing and expansion microscopy, have enabled super resolution imaging of the neural circuits containing thousands of neurons at a single synapse resolution. The imaging resolution and volume achieved by new technologies are beyond the limits of conventional light or electron microscopic methods. Progress in genome editing and related technologies has made it possible to label and manipulate specific cell types and discriminate activities of multiple cell types. These technologies will provide a breakthrough for multiscale analysis of the structure and function of local neural circuits. This review summarizes the basic concepts and practical applications of the emerging technologies and new insight into local neural circuits obtained by these technologies.

## Introduction

The neural network in the brain can be considered an integrated system consisting of many local circuits with particular functions. For this reason, proper computation in local neural circuits and their efficient interactions within and between cortical areas are essential for information processing in the vertebrate cortex. Studies on local neural circuits also provide new insights into the etiology of brain diseases. This approach is particularly effective in the research of psychiatric disorders, which are postulated to be caused by neural circuit dysfunctions in the human neocortex and subcortical structures.

The mammalian neocortex is organized into six layers normal to the cortical surface. Tangential to the cortical surface, it is also differentiated into multiple areas based on their specific inputs and outputs, such as sensory, motor, and association areas. Cortical regions are postulated to be organized into the assembly of smaller-scale local neural circuits. They are often defined as a neural circuit within a cylindrical volume 10–100 microns in diameter (Yoshimura et al., 2005; Perin et al., 2011; Molnár and Rockland, 2020). Local neural circuits can be identified across all cortical layers with varying properties in their intrinsic connectivity, scale, and interaction with other local circuits. Accordingly, local circuit analysis aims at clarifying two important aspects. First, how neurons within a local neural circuit follow the principles of connectivity based on their cell type, morphology, gene expression, and neural activity. Second, how the input-output characteristics of local neural circuits determine the region-specific cortical functions, such as motor control and sensory perception, which are critical in the control of animal behavior.

**Figure 1:**
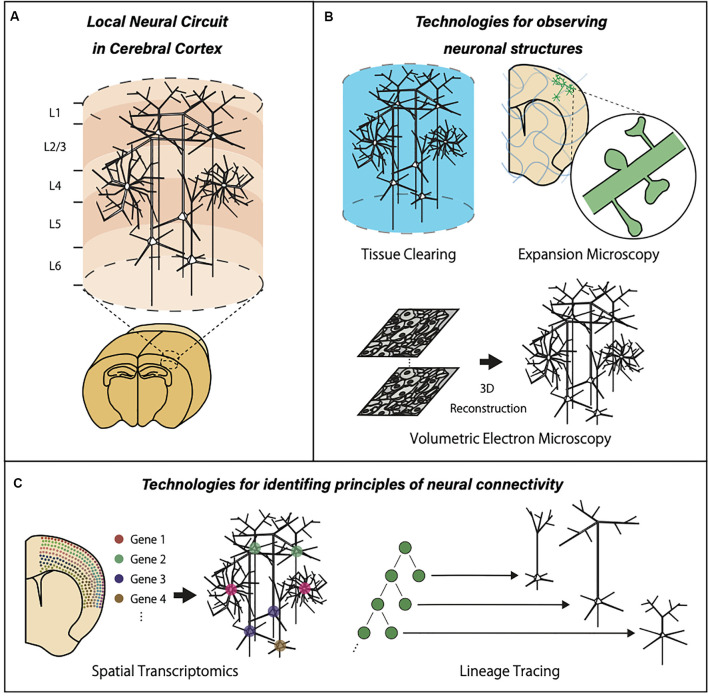
Graphic summary of neurotechnologies for local neural circuits in the cerebral cortex. **(A)** The cerebral cortex is organized with many units of locally connected neurons extending the entire cortical layers. **(B)** Optical clearing methods, expansion microscopy, and a large-volume EM reconstruction allow three-dimensional observation of the large brain volume with subcellular resolution. **(C)** The advancements of spatial transcriptomics and cell lineage tracing provide valuable information about gene expression patterns and developmental events within local neural circuits.

To solve these two major questions, researchers will need novel technologies to break the limitations of currently available methods (Figure 1). One of the largest limitations in imaging-based analysis is a trade-off between resolution and field of view. Conventional electron microscopy (EM) provides sub-nanometer resolution with a limited tissue volume, while intrinsic optical imaging provides brain-wide activation patterns without cellular resolution. Therefore, new technologies that can image single neurons or synaptic structures with a large field of view should be developed. Optical clearing methods, expansion microscopy, and a large-volume EM reconstruction are new trends in neurotechnology aiming at the simultaneous observation of both local subcellular properties and global features of the neural circuits. Another limitation in the current neural circuit analysis is the lack of information about the previous biological history of individual neurons embedded in a circuit. This lack of information is especially problematic in two important cases. First, precise interpretation of learning-related circuit modification is impossible without information about activity-dependent transcriptional and translational regulation. This can be achieved only by combining recording of multicellular activity and comprehensive expression analysis of multiple mRNA/protein species, which requires advancement in spatial transcriptomics and related technologies. Second, the establishment of neural circuits requires proper proliferation, migration, process extension, and connection of individual neurons. This developmental process is intimately linked to the final architecture of the neural circuits. Therefore, the identification of the principles in neural connectivity requires knowledge about the previous developmental events and temporal sequences of cellular differentiation. To obtain information about developmental events, accurate and extensive cell lineage tracing should be combined with neural circuit analysis.

In summary, progress in neural circuit analysis requires integration with multiple novel technologies based on imaging, tissue processing, transcription analysis, and cell lineage tracing. This review introduces the latest technologies essential in local neural circuit studies, mainly from reports focusing on the mouse cerebral cortex. Furthermore, we will discuss the new data and models of local neural circuits using such innovative techniques.

### Optical Clearing

The structure of neural circuits at the single-cell and subcellular levels has been studied traditionally by observing neural morphology under an optical microscope after preparing thin brain sections. This technique helps classify cell types based on morphology and estimate the connectivity of neural circuits. However, the opaqueness of biological tissues limits the observation to a depth of tens of microns. The recent innovation in two-photon and multi-photon microscopes achieved imaging of fluorescent objects located more than 1 mm deep inside brain tissues. Still, it is challenging to perform whole-body and whole-organ imaging even with the currently developing non-linear optical microscopic techniques.

Researchers developed methods of increasing tissue transparency to overcome the limitation in the imaging depth of fluorescent samples. The technique also helps researchers obtain information about 3D tissue organization. Tissue clearing methods reduce light scattering and absorption in tissue specimens and facilitate in-depth observation, which has been difficult with the conventional technique. Tissue transparency can be increased by removing lipids, which are the main factor of light scattering *in vivo*, and by matching the refractive index (RI) of the solvent to that of the tissue. To date, there are three major strategies for tissue clearing: methods using hydrophobic reagents, methods using hydrophilic reagents, and hydrogel-based techniques.

Clearing with hydrophobic reagents is achieved by organic solvents with a high RI (Ertürk et al., 2012; Renier et al., 2014; Pan et al., 2016; Cai et al., 2019). The penetration of organic solvents requires dehydration that results in fluorescence quenching, together with the shrinkage and hardening of samples. However, dehydration is beneficial in making samples with high transparency and suitable for long-term storage. New methods using immunostaining, nanoparticles, antioxidants, and nanobodies have been proposed to overcome the quenching problem (Renier et al., 2014; Pan et al., 2016; Cai et al., 2019). Transparent brain tissue generated by hydrophobic reagents could visualize long-range axonal projections in the brain (Schwarz et al., 2015; Lin et al., 2018).

Tissue clearing based on hydrophilic reagents removes lipids using surfactants and adjusts the RI of clearing solutions to minimize RI mismatches at interfaces. Major protocols of hydrophilic reagents are Sca*l*e (Hama et al., 2011), SeeDB (Ke M. T. et al., 2013), and CUBIC (Susaki et al., 2014). Sca*l*e is a method using a hydrophilic reagent containing urea, glycerol, and a detergent (Hama et al., 2011). Urea can improve the hydrophilicity of specimens without quenching fluorescence. Delipidation with the detergent, milder than that with organic solvents, is advantageous for preserving fine morphology. Preservation of fine morphology with Sca*l*e was evaluated in brains expressing YFP in a subset of hippocampal and cortical neurons. Sca*l*e-treated samples were suitable for high-resolution imaging that permitted visualization of the dendritic spines in layer 5 pyramidal neurons. Furthermore, Sca*l*e allowed researchers to perform two-photon microscopy of deep brain structures such as the hippocampus from the brain surface through all cortical layers. Original Sca*l*e protocol was not optimized for tissue preservation and could not prevent tissue swelling associated with urea treatment. Sca*l*eS, an improved version of the Sca*l*e method, was subsequently invented to solve these problems (Hama et al., 2015). In the Sca*l*eS protocol, samples were treated with a solution containing urea and sorbitol. Because no detergent was used in Sca*l*eS, the integrity of membrane organelles was sufficient for *post hoc* electron microscopic observation. Accordingly, electron microscopic examination of presynaptic active zones and amyloid plaques in the Alzheimer’s disease model was possible (Hama et al., 2015). Sca*l*eSF has recently been proposed as an improved method of Sca*l*eS to enable correlative light and electron microscopy (Furuta et al., 2021).

See Deep Brain (SeeDB) was developed as an optical clearing method optimized to maintain fine-scale cell morphology (Ke M. T. et al., 2013). SeeDB treats brain tissues with fructose, a high refractive index solution, to increase transparency. SeeDB can preserve structural details in biological samples, mainly because of the absence of detergents, which cause tissue degeneration in other clearing methods. The extent of tissue clearing in SeeDB is sufficient to image the hippocampal dentate gyrus through the entire cortex by conventional one-photon microscopy. Another advantage of SeeDB is compatibility with lipophilic tracers, such as DiI. SeeDB was later proposed to be useful for superresolution imaging in combination with Iohexol, a reagent for increasing the refractive index of solutions, and a mild detergent of saponin. This improved SeeDB was named SeeDB2 (Ke et al., 2016). SeeDB2 allows superresolution imaging to deep tissues without noticeable subcellular damage, which is sometimes problematic in other optical clearing methods. Although SeeDB2 may not be suitable for whole-brain imaging, it is superior in maintaining the microstructure. Ke and his colleagues investigated the fine-scale structure of dendritic spines in layer 5 pyramidal neurons in the cerebral cortex of NMDAR KO mice treated with SeeDB2 (Ke et al., 2016). Other research groups analyzed the PSD-95-positive postsynaptic densities in thorny excrescences and Bassoon-positive active zones in mossy fiber terminals in the hippocampal CA3 using SeeDB2 (Weng et al., 2018).

The development of an imaging pipeline using tissue clearing samples is highly beneficial for researchers studying cell and tissue organization on a large-scale. A modified Sca*l*e method combined with imaging protocols and an analytical pipeline was proposed for whole-organ imaging with single-cell resolution. The package, called CUBIC, contains an efficient tissue clearing protocol, high throughput imaging using light-sheet microscopy, and automated 3D analysis to compare different samples quantitatively (Susaki et al., 2014). The clearing protocol in CUBIC includes the following reagents: N-butylethanolamine and 1,3-Bisaminomethylcyclohexane for delipidation and decolorization; antipyrine and N-methyl nicotinamide for refractive index adjustment; and EDTA for demineralization. CUBIC allows whole-brain imaging with single-cell resolution using a light-sheet microscope in about an hour. Mice expressing degradable Venus fluorescent protein under the control of an activity-dependent Arc promoter was analyzed by CUBIC, which identified neurons in response to light stimulation within the entire brain. CUBIC reagents have also been used to analyze the morphology and molecular profiles of cortical astrocytes (Lanjakornsiripan et al., 2018) and to map long-range axons in the whole brain (Economo et al., 2016; Winnubst et al., 2019).

In hydrogel-based clearing methods, the tissue is cross-linked with polyacrylamide gel after fixation, and then SDS electrophoresis is performed to remove lipids. This protocol was named CLARITY (Chung et al., 2013). In this technique, macromolecules in tissues are cross-linked to the hydrogel polymers by paraformaldehyde. Lipid-containing structures, such as plasma membranes and myelin, are efficiently eluted, while biomolecules remain attached to hydrogels. CLARITY can achieve high transparency and high antibody penetration compared to other tissue clearing methods. The stability of biomolecules in the hydrogel allows effective antibody removal by SDS and repetitive immunolabeling. CLARITY has been used for wide-field imaging of neural circuits (Ye et al., 2016) and 3D imaging of RNA distribution (Sylwestrak et al., 2016). For better tissue preservation, a modified method (passive CLARITY/PACT) has also been proposed (Tomer et al., 2014; Yang et al., 2014). Passive CLARITY/PACT combines lower polymer density with passive delipidation, a process of SDS buffer exchange without electrophoresis. CLARITY is demanding in its setup and procedural complexity compared to other clearing methods. Another issue is fluorescence quenching by strong denaturing steps (Jensen and Berg, 2017).

To date, a wide variety of optical clearing methods have been proposed and applied to the analyses of microstructures in diverse organs and the tracing of long-range neurite projections (Ueda et al., 2020a; Tian et al., 2021). However, since each clearing method has its suitable applications, careful selection of the protocol is necessary, according to the required imaging volume, resolution, and available fluorescent probes (Tainaka et al., 2016; Ueda et al., 2020b).

With recent advances in optical clearing techniques, 3D whole-brain image data can be obtained with subcellular resolution using optical microscopes. In the future, we will need ideas on how to utilize the obtained large data sets and extract useful information. One of the future directions is to reveal hidden patterns of tissue architecture and cell morphology by mathematical analysis and artificial intelligence. Another possibility is to integrate optical clearing techniques with cell typing based on advanced transcriptomic technology. New information about cell types integrated into local circuit organization will reveal its building principle. There will be an increase in studies aiming to integrate imaging data obtained by optical clearing methods with physiological data acquired by electrophysiological recordings, *in vivo* calcium imaging, time-lapse imaging, and interventions such as optogenetic manipulations.

### Expansion Microscopy

The efficient computation in the brain is based on the proper organization of local circuits. The full description of local neural networks requires detection and measurement of neuronal nanostructures, including dendrites, axons, and synapses. The resolution of conventional optical microscopy is limited to the diffraction limit. Although superresolution microscopy can break the diffraction limit, it requires a high local dose of light for fluorophore excitation (Sahl et al., 2017) and is difficult to be applied to the volume imaging of local neural circuits. Volume imaging of nanoscale tissue structure requires a new design of tissue imaging that allows the increase of effective resolving power in combination with a low-dose fluorescence imaging scheme. Expansion microscopy (ExM) was proposed to overcome the resolution limit of conventional optical microscopy by physically expanding tissue specimens (Chen F. et al., 2015). In this method, water-absorbing polymers, such as Acryloyl-X, are embedded inside samples of chemically fixed cultured cells or brain sections. Acryloyl-X covalently binds to the amine groups of proteins and preserves the localization of antigens after digestion with proteinase K. Subsequent addition of water initiates isotropic expansion of the specimen with minimal deformation. The first study of ExM reported ~4× linear expansion of the samples with an effective resolution of ~70 nm, comparable to superresolution microscopy. ExM achieved visualization of clathrin microstructure and the alignment of pre- and postsynaptic membranes (labeled by Homer1 and Bassoon). The distances between neighboring Homer1 puncta and Bassoon puncta in ExM samples were compatible with that measured by structured illumination microscopy (SIM). They also reported superresolution volumetric imaging of synaptic organization within 500 μm × 180 μm × 100 μm of mouse brain sections. These results suggest that ExM is an advantageous technique to visualize the nanoscale details of complex three-dimensional structures such as local neural circuits.

Various applications of ExM technology have been reported to date. One such example demonstrated superresolution imaging in combination with light-sheet illumination for data acquisition from a large tissue volume (Bürgers et al., 2019; Gao et al., 2019). Image acquisition in millimeter-scale ExM samples by scanning microscopy requires a long time and may be compromised by fluorescence bleaching. ExM samples are transparent and suitable for illumination with a light sheet. Light-sheet microscopy enables us to acquire 3D imaging data from a large volume of ExM samples.

In light-sheet microscopy, excitation light is shaped into a thin sheet and applied to the sample perpendicularly to the direction of observation (Dodt et al., 2007; Chen et al., 2014). In the ExM preparation of mouse cortical sections, light-sheet microscopy could image synapses at a resolution of 60 nm × 60 nm × 90 nm. The acquisition was completed ~40 times faster than conventional laser scanning microscopy (Gao et al., 2019). In another report, correlative imaging between *in vivo* two-photon calcium imaging and ExM with synaptic markers was demonstrated (Lee et al., 2019). After *in vivo* two-photon calcium imaging in dendritic spines in mouse visual cortex, the authors identified the same spines using a modified version of ExM, ×10 ExM (Truckenbrodt et al., 2018; Lee et al., 2019). This correlative imaging method discovered that nearby spine pairs, each receiving input from different sources, exhibited similar orientation selectivity. A variety of techniques have been successfully combined with ExM, including proteomic analysis achieved by multiple immunostainings (Ku et al., 2016), connectome analysis of neurons with specific molecular profiles (Shen et al., 2020), and transcriptomics (Chen et al., 2016; Alon et al., 2021).

The application of ExM flexibly combined with other techniques is beneficial for neuroscience (Karagiannis and Boyden, 2018; Gallagher and Zhao, 2021). One possibility is the integration of learning-related behavioral analysis combined with synapse imaging and ExM. Furthermore, recent *in vitro* studies revealed the relationship between activity-dependent regulation in synaptic efficacy and dynamic remodeling of synapse nanostructure as a critical component in the regulation of neural circuits (Okabe, 2020a; Obashi et al., 2021). Therefore, identification and nanoscale imaging of synapses involved in learning-related circuit alterations *in situ* using ExM will benefit the research program of functional neural circuits.

Researchers should be aware of several limitations in ExM (Karagiannis and Boyden, 2018). First, the multiple steps of sample preparation in ExM require careful handling of the specimens, which are fragile and highly transparent. Second, the enlargement of specimens by ExM decreases the density of fluorescent molecules, resulting in a lower fluorescence signal. Third, expansion of imaging volumes inevitably increases the data size. Therefore, it is necessary to evaluate whether ExM is beneficial for specific research subjects carefully. The research group initially developed ExM have provided detailed information about the technology and tips for application, and attempts have been made to increase the versatility of ExM (Gao et al., 2017; Yoon et al., 2017; Wassie et al., 2019).

### Volumetric Electron Microscopy

In classical observation with optical microscopy, two neurons were judged to be connected by putative synapses if axonal boutons and dendritic spines overlapped (Hill et al., 2012). However, the proximity of axons and dendrites is not a reliable indicator of synaptic connections (Mishchenko et al., 2010; Briggman et al., 2011; Helmstaedter et al., 2013; Okabe, 2020b). The definite identification of synaptic connection requires the apposition of presynaptic active zones and the postsynaptic membrane thickenings (postsynaptic density), which can be unambiguously identified only by electron microscopy. The wiring diagram of local neural circuits can be generated if structures of all neurons and their synaptic junctions to other neurons are detected and recorded by electron microscopy. The complete 3D reconstruction of a tissue volume and comprehensive identification of all synaptic connectivity referred to as “connectome”, is a technically demanding but highly valuable research subject in neuroscience (Morgan and Lichtman, 2017; Kornfeld and Denk, 2018).

Electron microscopy is a technique that achieves imaging resolution much higher than light microscopes. There are two types of electron microscopes. In a scanning electron microscope (SEM), samples are irradiated with a focused electron beam, and raster images are generated by detecting secondary or backscattered electrons from the irradiated surface. In a transmission electron microscope (TEM), images are generated by the interaction of an electron beam with ultrathin sections and captured by a high-sensitive camera, CCD, or cMOS camera. The electron microscopic images of nervous tissues have revealed nanoscale structures such as synaptic clefts, synaptic vesicles, and postsynaptic thickenings, which are involved in the process of neurotransmission (Gray, 1959; Akert et al., 1967).

For reconstruction of local neural circuits by electron microscopy, a large number of serial images have to be taken, and the obtained images have to be aligned to reconstruct in 3D. Moreover, the 3D images have to be computationally segmented and labeled as individual components (Smith, 2007; Briggman and Bock, 2012). A pioneering study in the 3D reconstruction of the whole nervous system was performed using a model animal, *Caenorhabditis elegans* (White et al., 1986). These early structural studies of *C. elegans* were achieved by the collaboration of Sydney Brenner, John Sulston, and John Graham White. However, without efficient and automated technology to process large volume samples in the 1980s, large-volume EM reconstruction was only possible by manual preparation of a large number of serial ultrathin sections. Therefore, many researchers have challenged in establishing platforms for the large-volume reconstruction of neural circuits by electron microscopy (Kornfeld and Denk, 2018; Sanes and Zipursky, 2020).

There are two approaches to volume imaging in electron microscopy. In the ultrathin section approach, ultramicrotome-based serial sectioning is first performed to generate and collect multiple ultrathin sections, which are observed by either TEM or SEM to produce serial section electron micrographs. In the serial block-face approach, imaging of the sample surface and removal of a thin surface layer were coupled and repeated multiple times to obtain serial images. The ultrathin section approach is commonly used for TEM-based reconstruction (Harris et al., 2006). Sample preparation for serial sections follows the conventional protocols for TEM. Preparing a large number of serial sections is time-consuming and labor-intensive (Harris et al., 2006; Bock et al., 2011; Lee et al., 2016). Some groups have challenged to streamline these processes (Tapia et al., 2012; Lee et al., 2018). An automated tape-collecting ultramicrotome scanning electron microscope (ATUM), which automatically collects ultrathin sections (<50 nm in thickness) on a plastic tape, has been developed to produce a large number of continuous ultrathin sections (Hayworth et al., 2006, 2014; Schalek et al., 2012). Because tapes used in ATUM are not transparent to an electron beam, SEM-based data acquisition is necessary for ATUM (Kasthuri et al., 2015). The advantage of the serial section approach is that sections can be re-imaged, and molecules in tissue sections can be identified by immunostaining before EM analysis (Kasthuri et al., 2015). For the serial section approach, a parallelized platform using multiple TEMs has also been developed (Yin et al., 2020).

The block-face approach is based on one of the following two SEM-based systems: the serial block face-SEM (SBF-SEM; Denk and Horstmann, 2004) or the focused ion beam-SEM (FIB-SEM; Heymann et al., 2006; Knott et al., 2008). SBF-SEM performs repetitive sectioning of the sample block surfaces with a thickness of ~20 nm. In FIB-SEM, the focused ion beam first scans and sputters a very thin surface layer of the sample block (in a range of few nanometers) to expose a new surface, which is re-scanned by the second standard electron beam for imaging. For this reason, the block-face approach has better resolution in the z-direction than ATUM (Knott et al., 2008; Briggman et al., 2011). In addition, the alignment step of serial SEM images for 3D reconstruction is less problematic because of the smaller amount of image drift and deformation between adjacent images in comparison with the ultrathin section approach (Kubota et al., 2018). However, this approach does not allow re-imaging of the surface later, nor does it permit prior immunostaining to analyze molecular distribution.

Segmentation and annotation of biological structures from serial EM images are the bottlenecks of the connectome. Therefore, there is a growing interest in developing techniques to automate reconstruction and improve the accuracy of image segmentation (Kornfeld and Denk, 2018; Moen et al., 2019). Various types of software have been developed to annotate microstructure, including neuronal processes and synapses from serial z-stack images (Fiala, 2005; Saalfeld et al., 2009; Helmstaedter et al., 2011; Cardona et al., 2012). Manual segmentation of a large sample volume is time-consuming and labor-intensive. Even though the software has been developed (Sommer et al., 2011; Kaynig et al., 2015) to support manual segmentation, *post hoc* correction of the segmented data is still required depending on the image quality. Automated segmentation tools incorporating machine learning have been proposed to overcome the difficulty (Berning et al., 2015; Januszewski et al., 2018). The machine learning algorithms in these tools are based on convoluted neural networks with modifications to minimize the error rate of tracing (LeCun et al., 2015). Manually annotated small data sets are used for training the neural network, which can even outperform manual segmentation. An automated approach is advantageous in both speed and accuracy. A mouse cortical region (93 μm × 60 μm × 93 μm) was segmented and annotated ten times faster than manual analysis (Berning et al., 2015). The neural connection in an adult zebra finch (about 1 million μm3) was reconstructed with high accuracy (Januszewski et al., 2018).

Electron microscopic data acquisition and circuit reconstruction are essential in comprehensive neural circuit analysis, which contributes to identifying organization principles of local neural circuits. Next-generation electron microscopic technology combined with *in vivo* functional studies will increase their value in neuroscience by revealing the relationship between circuit structure and its function (Luo et al., 2018). However, designing and building electron microscopes with a large imaging field and high image acquisition speed, together with sample preparation equipment, demand the active collaboration of biology and engineering. So far, few platforms rapidly and accurately complete the connectome of the entire mouse cortex. In this section, we focus on the research of local neural circuits in the mammalian cerebral cortex. EM volume imaging is a rapidly advancing technology, and its targets in neuroscience and other research fields are increasing rapidly (Smith, 2007; Briggman and Bock, 2012; Kubota, 2015; Xu et al., 2017; Kornfeld and Denk, 2018; Kubota et al., 2018).

### Spatial Transcriptomics

Local neural networks contain multiple neuron types, which show gene expression patterns correlated with their specific functions and connectivity. For example, various types of interneurons in the mammalian cortex express particular gene sets, including calcium-binding proteins, neuropeptides, and molecules involved in the formation of specific synaptic connections (Zeisel et al., 2015). Apart from the differential gene expression in distinct cell types, experience-dependent gene expression and epigenetic regulation associated with inflammation and environmental stress are critical factors in the proper development and functional maturation of cortical neural circuits. Therefore, comprehensive gene expression data provides indispensable knowledge about the differentiation of cell types, experience-dependent circuit modification, and the brain’s stress responses.

*In situ* hybridization (ISH) is a method to determine gene expression patterns in tissues by hybridizing the labeled probes to the target mRNAs in whole tissues and sections (Lein et al., 2007). Both fluorescent probes and enzyme-coupled probes are used for visualization (Femino et al., 1998). An increase in the number of target genes in ISH is essential in transcriptomic data analysis. Improvement in spectrally resolvable fluorescent probes and sequential imaging protocols contributed to the progress of multiplexing fluorescence ISH (FISH). Multiple rounds of imaging, probe removal, and probe rehybridization enable the detection of a large number of target transcripts (Lubeck et al., 2014). There is significant progress in sequential FISH technology (Chen K. H. et al., 2015; Codeluppi et al., 2018; Shah et al., 2018; Eng et al., 2019), which has been applied to the mouse cortex (Codeluppi et al., 2018) and hippocampus (Shah et al., 2016, 2017).

*In situ* sequencing (ISS) is a method of sequencing mRNA directly in cell samples or fixed tissues. Local sequencing of transcripts requires amplified cDNAs tethered to the sample tissue. In ISS protocols, RNA molecules are chemically tethered to tissues, reverse-transcribed, and amplified extensively into submicron-sized DNA nanoballs using padlock probes combined with rolling circle amplification (Lizardi et al., 1998; Ke R. et al., 2013; Lein et al., 2017; Asp et al., 2020). The padlock probe contains complementary sequences to target RNA and short barcode sequences. Therefore, the barcode sequences were also amplified *in situ* together with the target gene. After clonal amplification of cDNAs, fluorescence-based sequencing technology is applied to individual DNA nanoballs. Sequencing chemistry is based on repetitive hybridization and ligation of color-coded oligoprobes. Either a short barcode inside the padlock probe or endogenous RNA molecules can be sequenced. ISS is applied to a variety of biological samples (Lee et al., 2014; Chen et al., 2018; Gyllborg et al., 2020), combined with other tissue-processing techniques (Wang et al., 2018), and shown to be compatible with ExM for gene expression analysis with subcellular or single spine resolution (Alon et al., 2021).

Both ISH and ISS are techniques for the *in situ* detection of RNA molecules. An alternative strategy to achieve spatial transcriptomics is *ex situ* sequencing of cDNAs containing both tissue-derived RNA sequences and small barcodes for spatial coordinates. This method, *in situ* capturing technology, uses a patterned microarray for capturing target RNA molecules (Asp et al., 2020). The microarray has spots with oligo dT probes with specific barcode sequences for the x-y coordinates of the glass slide. A tissue section attached to the glass slide is permeabilized to release mRNAs, which are captured to the nearest spots of oligo dT probes. After reverse transcription and sequencing, information of both mRNA species and the x-y coordinates can be obtained. The spatial resolution, which depends on the size of the spots, was not sufficient for single-cell resolution in the initial study (Ståhl et al., 2016). However, the incorporation of microbeads to capture mRNAs improved spatial resolution subsequently (Vickovic et al., 2019; Stickels et al., 2021). Furthermore, reconstructing serial brain sections on the patterned microarray produced the 3D map of multiple transcripts (Rodriques et al., 2019). Application of this technology to the mouse model of Alzheimer’s disease was also reported (Chen et al., 2020).

ISH, ISS, and *in situ* capturing technology are suitable for high throughput gene expression analysis with a high spatial resolution (Waylen et al., 2020; Ortiz et al., 2021). At present, several limitations exist in these technologies. Multiplexed ISH and ISS require special equipment for sequential fluorescent labeling and imaging. Sample distortion and drift during hybridization and sequencing are problematic, especially in image registration to match fluorescent spots corresponding to the amplified transcripts. *In situ* capturing technology is a labor-intensive method due to its necessity of handling huge transcriptomic data. Various platforms have been proposed for the efficient analysis of large data sets (Edsgärd et al., 2018; Cang and Nie, 2020; Svensson et al., 2020; He et al., 2021).

Spatial transcriptomics technology is a powerful tool linking gene expression in single neurons to their functional and structural characteristics within local neural circuits. Previous neurophysiological experiments indicated that the probability of synaptic connections between neurons is regulated by the neuron subtypes. Furthermore, the location of synapses along dendrites has been shown to be cell-type specific. Thus, extensive transcriptomic analysis of individual neurons combined with the study of their connectivity will both promote a more precise classification of neurons and their circuit-level functions.

### Lineage Tracing

Imaging neuronal morphology and activity provides valuable information about the existing neural circuits. However, understanding the design principles of constructing neural circuits requires methods of identifying the previous history of cell proliferation, migration, and differentiation. Diverse neuronal subtypes contribute to the formation of local neural circuits during development (Holguera and Desplan, 2018; Yuste et al., 2020). How cell lineage and neuronal diversity are linked to the formation of functional neural networks is a central question in developmental neurobiology.

Cell lineage tracing identifies the daughter cells derived from a single parental cell in developing tissue (Kretzschmar and Watt, 2012). In neuroscience, this technique is applied to identify single cell-derived clones within neural circuits. Traditionally, retroviral tracers, including vectors derived from Molony Murine Leukemia Virus (MMLV), have been used to identify single cell-derived clones in developing nervous tissues (Sanes et al., 1986; Turner and Cepko, 1988). Retroviral tracers infect only proliferating cells and are integrated into the host genome after reverse transcription of the viral RNA. The integrated virus genome kept inside daughter cells enables lineage tracing. Retrovirus-mediated cell lineage tracing identified clonally related cortical excitatory neurons within a columnar unit (Rakic, 1988) with functional synaptic connections (Yu et al., 2009) and similar stimulus responsiveness (Li et al., 2012). Conversely, single clone-derived interneurons form electrical synapses (gap junctions) rather than chemical synapses (Li et al., 2012). These results suggest that cell lineage plays a key role in establishing synaptic connections. Although lineage tracing by retroviruses has been used widely, this technique also has limitations. For example, genome integration is random, which leads to silencing or mutation of endogenous genes (Panganiban, 1985). The weakness of reporter gene expression in retrovirus-infected cells is another concern. These limitations have been improved by introducing insulators (Antoniou et al., 2013) or expressing retroviral receptors on specific cells (Doetsch et al., 1999).

Mouse molecular genetics has been contributed in cell lineage tracing by knock-in of reporter genes (Mao et al., 1999; Srinivas et al., 2001) and genetic mosaic mice expressing several fluorescent proteins with Cre/LoxP recombination (Zong et al., 2005; Livet et al., 2007; Muzumdar et al., 2007; De Gasperi et al., 2008; Cai et al., 2013; Loulier et al., 2014). Time-specific conditional recombination is a powerful approach to cell lineage tracing. A representative example of a temporally regulated gene recombination system is Mosaic Analysis with Double Markers (MADM; Zong et al., 2005). MADM is based on restoring the intact GFP or RFP coding sequences after Cre-dependent interchromosomal recombination at specific developmental time points. After inducing Cre-activity, a small fraction of proliferating cells shows interchromosomal recombination to restore both GFP and RFP expression cassettes in respective alleles. These restored GFP and RFP gene cassettes may either segregate into two daughter cells to generate two cells with distinct colors or go to one of the daughter cells to generate a single double-colored cell and an unlabeled cell. Thus, MADM can label two daughter cells from a single parental cell with two different fluorescent proteins in a stochastic fashion (Zong et al., 2005; Tasic et al., 2012). MADM revealed relationships between neural activity and cell lineage in the cerebellum (Espinosa and Luo, 2008), hippocampus (Xu et al., 2014), cerebral cortex (Gao et al., 2014; Beattie and Hippenmeyer, 2017; Cadwell et al., 2020), and hypothalamus (Zhou et al., 2020).

Lineage tracing can be performed by light microscopic detection of cells expressing lineage markers. Sparse labeling of proliferating neural progenitors results in spatial isolation of single cell-derived clones and facilitates cluster identification by microscopic examination. However, if labeled neural progenitors show extensive migratory behaviors, as in the case of cortical interneurons, identification of widely dispersed clones needs an additional strategy. Furthermore, to achieve parallel identification of multiple single cell-derived clones by dense labeling, additional techniques to distinguish individual clones are required. By generating a library of retrovirus vectors carrying heterogenous DNA fragments with their complexity of several hundred, brain-wide dispersion of single cell-derived clones could be detected. Initial experiments of identifying dispersed clones in the brain required microdissection of labeled neurons and subsequent PCR amplification of the marker DNA for restriction enzyme mapping (Walsh and Cepko, 1992; Reid et al., 1995). Recent advances in single-cell DNA sequencing facilitate comprehensive identification of single cell-derived clones dispersed in the entire brain (Woodworth et al., 2017; Kester and van Oudenaarden, 2018; Wagner and Klein, 2020).

Theoretically, cell tracing experiments can be grouped into two different strategies. The first strategy is prospective tracing, in which cells in the early developmental stage are genetically marked, and their fate in later developmental stages is analyzed. Both retroviral tracing and transgene-based mosaic analysis are grouped in this category. The second strategy is retrospective tracing, which utilizes the somatic mutations present in mature organs for tracing cell lineage. Somatic mutations occur with low frequency throughout the development and permanently mark the progeny of the initially mutated cell. If efficient identification of somatic mutations in single cells is possible, reconstruction of the lineage tree of multiple cells can be achieved. Although conceptually attractive, this strategy is technically demanding due to the low frequency of naturally occurring somatic mutations and the requirement of whole-genome sequencing of single cells. The introduction of CRISPR/Cas9 genome-editing technology has recently been shown to help retrospective tracing of cell lineage. The Genome Editing of Synthetic Target Arrays for Lineage Tracing (GESTALT) introduces multiple unique and accumulating mutations into artificial barcode sequences containing multiple CRISPR/Cas9 target sites (McKenna et al., 2016). When multiple barcode sequences are introduced into the zebrafish genome and fertilized eggs were injected with editing reagents targeting the barcodes, uniquely edited barcodes were progressively generated over cell divisions, and cell lineage relationships could be reconstructed based on the mutational pattern (McKenna et al., 2016). This study demonstrates the potential of retrospective tracing in developmental biology and accelerated the development of similar CRISPR/Cas9 system-based cell lineage tracing methods (Perli et al., 2016; Shipman et al., 2016; Kalhor et al., 2017; Alemany et al., 2018; Spanjaard et al., 2018; Garcia-Marques et al., 2020). In addition to cell lineage analysis, systems have also been proposed to record other biological events, such as transcriptional activity in the past (Farzadfard and Lu, 2014; Perli et al., 2016; Sheth et al., 2017; Tang and Liu, 2018).

Local neural circuits have not yet been the target of CRISPR/Cas9 system-based retrospective tracing. Before its application to neural circuits, several problems should be solved, such as sequence loss caused by double-strand breaks, non-specific sequence editing by Cas9, and insufficient spatial resolution required for the analysis of neural circuits. Gene-editing technologies are developing rapidly, with the introduction of new concepts, such as prime editing without double-strand breaks and base editing technology (Kantor et al., 2020). In addition, the technology of retrospective tracing of single cells *in situ* has been reported (Frieda et al., 2017; Raj et al., 2018).

These technological advancements will help neuroscientists establish reliable and simultaneous recording of gene expression patterns and cell lineage within local neural circuits. With the progress of lineage tracing technology, a neuronal population derived from a single radial glial cell will be identified and classified into subgroups according to their timing of differentiation and gene expression profiles. This progress in technology helps a more precise and comprehensive description of connectivity between neurons with their lineage identification.

## Applications

This section introduces three studies of local neural circuits in the cerebral cortex using the technologies described in the previous sections. The first example described in this section approaches the developmental perspective in the generation of repetitive neuronal cell assembly in the neocortex. A combination of tissue clearing technology and cell lineage analysis will become essential in this type of analysis. The second application is spatial transcriptomics of cortical neurons after tissue expansion. Localized translation in dendrites and axons is critical in neural network development and function, but the comprehensive analysis of both mRNA localization and neuronal connectivity has not yet been achieved. Progress in higher resolution analysis of mRNA distribution in association with local circuit function is necessary to fully understand experience-dependent circuit remodeling, which requires selective mRNA transport and local translation. The third example shows the power of EM-based dense circuit reconstruction in elucidating the principles of synaptic connectivity during postnatal cortical development. The efficiency of the EM-based connectome has increased rapidly to reach the stage of comparison of multiple data sets taken at different developmental stages. By sharing the EM connectome data in the community of neuroscientists, unexpected features and principles of neural circuits may emerge by a collaboration of biologists and data scientists. The following description of these three application help readers recognize the importance of transcending approaches in the progress of neuroscience.

Cellular organization in the mammalian neocortex has been studied intensively using neuroanatomical methods. The columnar organization of cortical neurons was discovered, and its importance in sensory information processing has been clarified (Molnár and Rockland, 2020). However, precise quantitative analysis in the spatial arrangement of columnar cell clusters has not yet been carried out. Is the arrangement of columnar cell clusters uniform across multiple cortical areas? Are cell types of cortical neurons related to the spatial organization of columnar cell clusters? Are there any lineage-specific properties in the columnar organization? To answer these questions, the data obtained by large-scale visualization of the cortical neuron distribution and neuronal cell lineage should be integrated.

**Figure 2:**
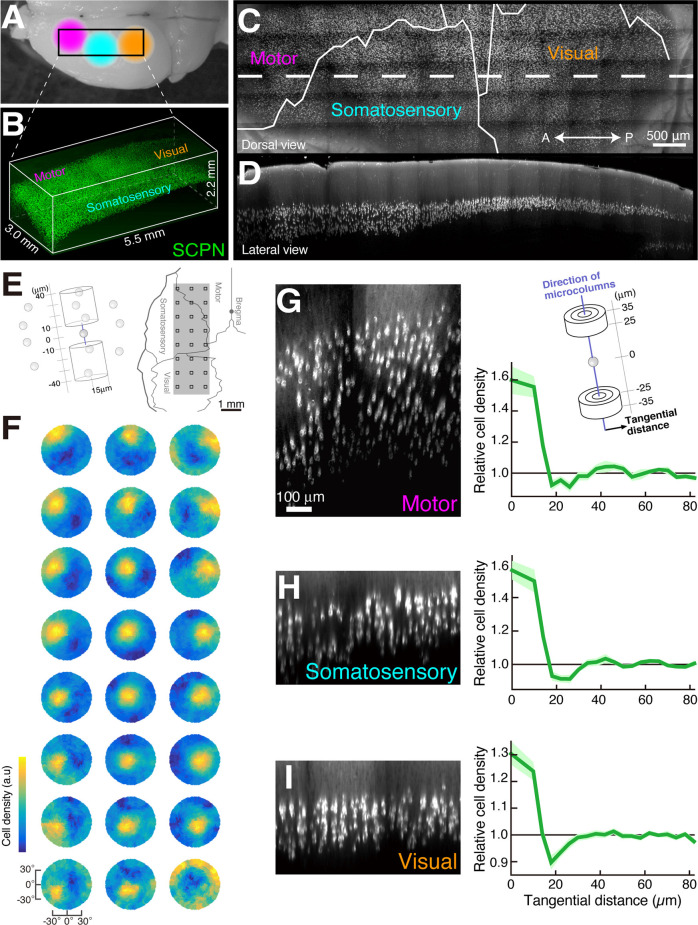
Microcolumns are present in multiple cortical areas (modified from Maruoka et al., 2017). Dorsal image of mouse brain **(A)** and 3D image data of subcerebral projection neurons (SCPNs, **B**). Dorsal image **(C)** and sagittal section image **(D)** of the white dotted area in **(C)** created from the image data in **(B)**. The determination of microcolumn axes **(E,F)**. We calculated the cell density of other SCPNs within two cylinders centered on the reference SCPNs (**E**, left). This calculation was performed within cylinders tilted at various angles, and the direction with the highest cell density was identified **(F)**. The squares in the right panel of **(E)** match the 3 × 8 data positions **(F)**. Cortical sections showing SCPNs **(G–I)** generated along the microcolumn axis identified in (**F**, left) and graphs showing the relationship between relative cell density of SCPNs and intercellular distance in the tangential orientation (perpendicular to the microcolumn axis). Cell densities were calculated in the two cylinders illustrated in **(G)**.

An optical clearing technique applied to the neocortex identified a highly organized spatial arrangement of layer 5 pyramidal neurons. Maruoka et al. labeled a major subtype of layer 5 pyramidal neurons and obtained the 3D coordinates of neuronal cell bodies in the mouse cortex after SeeDB treatment (Figure 2). The authors found that layer 5 neurons form subtype-specific microcolumns, and layer 5 is organized into repetition of those microcolumns (Maruoka et al., 2017; Yoneda et al., 2018). Based on this anatomical discovery, the authors further revealed that microcolumns operate as functional units of neural circuits. Interestingly, lineage tracing indicates that a single microcolumn is composed of sister neurons generated from different radial glial cells. In recent years, the advance of technologies such as transcriptomics has led to the detailed classification of neuronal subtypes in the cortical layers, including layer 5. Detailed classification of subtypes enables a new approach from anatomically identified 3D structures to the physiological property of local neuronal circuits.

Information about the gene expression of individual neurons is helpful in the identification of cell types and their developmental lineages. However, the spatially regulated transport of mRNAs and their local translation in both dendrites and axons have been shown to play a crucial role in the activity-dependent regulation of neural circuit remodeling. Therefore, it is necessary to develop new technologies that can record the positions of multiple mRNA species with high resolution inside the neuronal cytoplasm. A recent effort of combining the ExM technology and ISS-based spatial transcriptomics has led to the development of expansion sequencing (ExSeq; Alon et al., 2021). *In situ* sequencing followed by immunostaining allowed them to obtain gene expression profiles without disrupting the nanoscale spatial information within tissue samples. ExSeq successfully identified the positions of a large number of transcripts along the dendrites and even in the single spines of hippocampal CA1 pyramidal neurons. Some of the genes mapped by ExSeq included genes whose mRNA expression in dendrites was unannotated (such as Gabrg2, which codes GABAA receptor γ2 subunit). They also showed that ExSeq could be adapted to human specimens. For example, ExSeq could detect RNA in the nuclei of cancer cells derived from a biopsy sample with high spatial resolution and be effective in colocalization analysis of multiple cell types inside the tissue containing highly mixed tumor and non-tumor cells.

EM reconstruction provides a spatial resolution that can not be achieved by light microscopy. However, the technique is highly time-consuming and labor-intensive; thus, its application has been limited to the analysis of mature neural tissues. However, the recent improvement in both image acquisition, segmentation, and annotation of EM data has enabled researchers to analyze the developmental process of neural circuits from multiple 3D reconstruction data taken at different postnatal developmental stages. A recent study utilized SBEM to acquire 13 sets of electron microscopic data taken from the samples of the mouse neocortical L4 and L2/3 regions during development [postnatal days 5 (P5) to P56; Gour et al., 2021]. Large-scale reconstruction of the cortical volumes, 8.78 million um3 in total, was achieved by newly designed reconstruction tools (Boergens et al., 2017; Gour et al., 2021). This group focused on the formation process of local inhibitory circuits and extracted the circuit information from the dense reconstruction of single axons and synapses. The study indicated three types of inhibitory axons undergoing specific synaptic development (Gour et al., 2021). The first type is axons that preferentially form synapses in the apical dendrite from an early postnatal stage (~P5), and these synapses tend to be maintained during development. The second type is axons preferentially forming synapses in the cell body, which persist during development (P5–P28). The third type is axons preferentially forming synapses in the axon initial segments (AIS). These axons initially make synapses on a single AIS (P9–P14) but later show extensive remodeling to form multiple synaptic junctions (P28). This study showed that with the cutting-edge technology of EM reconstruction, temporal analysis of postnatal neural circuit development could be achieved by comparing dense reconstruction data at multiple time points. Comprehensive analysis of local neural circuits with EM reconstruction will lead to understanding universal principles of local neural circuits and quantitative insights into neural circuit function.

It should be emphasized that there are a number of new studies applying innovative technologies to neural circuit analysis; spatial transcriptome technology with optical clearing methods (Wang et al., 2018), a functional connectomics technology combined *in vivo* two-photon calcium imaging, and electron microscopy (Bock et al., 2011; Briggman et al., 2011; Lee et al., 2016; Scholl et al., 2021). Thus, the study of the local neural circuits has entered a new stage with the latest technical developments.

## Conclusion

In classical studies for local neural circuits, electrophysiological techniques have been the primary method, especially for the elucidation of connectivity. Electron microscopic observation has the potential to jump into the mainstream of research tools for local neural circuits. The optical clearing methods extend the observation to 3D wide-area imaging, and ExM breaks the limitation of optical resolution in light microscopy. Since computer technology has recently advanced to the point where petabyte-scale data can be processed, scientists have started applying artificial intelligence (AI)-based analysis to discover novel principles of local neural circuits. These research directions may open a way toward unexpected findings that conventional methodologies have overlooked.

Furthermore, advanced methods allow us to simultaneously obtain spatial expression profiles of a huge number of genes with subcellular resolution. Novel lineage tracing methods make it possible to read out the history of past intracellular events. It will soon become clear how gene expression profiles in individual neurons, including past intracellular events, correspond to the formation and function of local neural circuits.

## Author Contributions

All authors have made substantial, direct and intellectual contribution to this work. All authors contributed to the article and approved the submitted version.

## Conflict of Interest

The authors declare that the research was conducted in the absence of any commercial or financial relationships that could be construed as a potential conflict of interest.

## Publisher’s Note

All claims expressed in this article are solely those of the authors and do not necessarily represent those of their affiliated organizations, or those of the publisher, the editors and the reviewers. Any product that may be evaluated in this article, or claim that may be made by its manufacturer, is not guaranteed or endorsed by the publisher.
